# Pre-hospital advanced airway management by experienced anaesthesiologists: a prospective descriptive study

**DOI:** 10.1186/1757-7241-21-58

**Published:** 2013-07-25

**Authors:** Leif Rognås, Troels Martin Hansen, Hans Kirkegaard, Else Tønnesen

**Affiliations:** 1 Department of Research and Development, Norwegian Air Ambulance Foundation, P.O. Pox 94, 1441 Drøbak, Norway; 2 Department of Anaesthesiology, Pre-hospital Critical Care Team, Viborg Regional Hospital, Heibergs Allé 4, 8800 Viborg, Denmark; 3 Pre-hospital Critical Care Team, Aarhus University Hospital, Trindsøvej 4-10, 8100 Aarhus, Denmark; 4 Department of Pre-hospital Medical Services, Central Denmark Region, Oluf Palmes Allé 34, 8200 Aarhus, Denmark; 5 Centre for Emergency Medicine Research, Aarhus University Hospital, Trøjborgvej 72-74, Building 30, 8200 Aarhus, Denmark; 6 Department of Anaesthesiology, Aarhus University Hospital, Nørrebrogade 44, 8000 Aarhus, Denmark

**Keywords:** Pre-hospital, Out-of-hospital, Prehospital emergency care (MeSH), Emergency medical services (MeSH), Helicopter emergency medical service, Critical care (MeSH), Airway management (MeSH), Endotracheal intubation (MeSH), Difficult endotracheal intubation, Complications (MeSH), Patient safety

## Abstract

**Introduction:**

We report data from the first Utstein-style study of physician-provided pre-hospital advanced airway management.

**Materials and methods:**

Anaesthesiologists from eight pre-hospital critical care teams in the Central Denmark Region (a mixed rural and urban region with 1.27 million inhabitants) prospectively registered data according to the template for reporting data from pre-hospital advanced airway management. Data collection took place from February 1^st^ 2011 to October 31^st^ 2012. Included were patients of all ages on whom pre-hospital advanced airway management was performed. The objective was to estimate the incidences of failed and difficult pre-hospital endotracheal intubation, and complications related to pre-hospital advanced airway management.

**Results:**

The overall incidence of successful pre-hospital endotracheal intubation among 636 intubation attempts was 99.7%, even though 22.4% of pre-hospital endotracheal intubations required more than one intubation attempt. The overall incidence of complications related to pre-hospital advanced airway management was 7.9%. Following rapid sequence intubation, the incidence of first pass success was 85.8%, the overall incidence of complications was 22.0%, the incidence of hypotension 7.3% and that of hypoxia 5.3%. Multiple endotracheal intubation attempts were associated with an increased overall incidence of complications. No airway management related deaths occurred.

**Discussion:**

The overall incidence of successful pre-hospital endotracheal intubations compares to those found in other physician-staffed pre-hospital systems. The incidence of pre-hospital endotracheal intubations requiring more than one attempt is higher than suspected. The incidence of hypotension or hypoxia after pre-hospital rapid sequence intubation compares to those found in UK emergency departments.

**Conclusion:**

Pre-hospital advanced airway management including pre-hospital endotracheal intubation performed by experienced anaesthesiologists is associated with high success rates and relatively low incidences of complications. An increased first pass success rate following pre-hospital endotracheal intubation may further reduce the incidence of complications and enhance patient safety in our system.

## Introduction

### Background

Pre-hospital advanced airway management (PHAAM) is a potentially lifesaving intervention [1]. However, it carries a risk of serious complications that may threaten patient safety and worsen patient outcome [2-4]. The amount of published papers addressing pre-hospital airway management is substantial, but the results are difficult to interpret. This is partly due to large variations in the Emergency Medical Service (EMS) systems and Helicopter Emergency Medical Service (HEMS) systems involved, and partly because of differences in data recording and -reporting.

Although the airway management performance of physician-staffed EMS / HEMS [5-14] seems to be of a higher standard compared with that of paramedic-based systems [11,15], the risks and complications related to PHAAM in physician-staffed pre-hospital systems appear to be significant. The incidence of failed pre-hospital endotracheal intubation (PHETI) in physician-staffed EMS/HEMS is reported to be 1-2% by several authors [5-10,12,14,16,17] including the recent meta-analysis by Lossius et al. [11].

In London HEMS, the incidence of difficult PHETI requiring more than one attempt to secure a patent airway was 12.5% [14] and the incidences of different PHAAM-related complications are reported to be 5-10% [5,7-9,13,17-19] in different physician-staffed systems.

In 2009, Sollid et al. published a “Utstein-style” consensus-based template for reporting data from PHAAM [20]. The purpose of this template was “to establish a set of core data points to be documented and reported in cases of advanced pre-hospital airway management” [20]. No authors have until now published data collected in accordance with this template.

An international group of experts have recently named pre-hospital advanced airway management one of the top-five research priorities in physician-provided pre-hospital critical care [21]. No prospective studies have previously investigated PHAAM performed by anaesthesiologist in Danish EMS / HEMS; the quality of PHAAM performed in these services is therefore unknown.

### Objectives

The main objectives of the present study were to estimate the incidences of failed PHETI, difficult PHETI and PHAAM-related complications. We furthermore wanted to gain detailed knowledge about the patient population, the indications for PHAAM, the use of airway back-up devices and overall mortality.

## Materials and methods

### Study design

We designed a prospective descriptive study where we collected PHAAM-related data from our anaesthesiologist-staffed pre-hospital critical care teams according to the consensus-based template [20].

### Setting

The Central Denmark Region covers a mixed urban and rural area of approximately 13000 km^2^with a population of 1.27 million. The overall population density is 97.7 inhabitants pr. km^2^.

The standard EU emergency telephone number (1-1-2) covers all Denmark and there is an Emergency Medical Dispatch Centre in each of the five Danish regions. Emergency Medical Dispatch is criteria based.

The Central Denmark Region has a two-tiered EMS system. The first tier consists of 64 ground ambulances staffed with Emergency Medical Technicians (EMTs) on an intermediate or paramedic level (EMT-I / EMT-P). EMTs in The Central Denmark Region do not perform PHETI, nor do they use supraglottic airway devices (SADs).

The second tier consists of ten pre-hospital critical care teams staffed with an anaesthesiologist (with at least 4½ years’ experience in anaesthesia) and a specially trained EMT. Nine of the pre-hospital critical care teams are deployed by rapid response vehicles; the tenth team staffs a HEMS helicopter.

In the most rural parts of the region there are three rapid response vehicles staffed with an EMT and an anaesthetic nurse. The anaesthetic nurses do not use SADs nor do they perform Rapid Sequence Intubation (RSI) or other forms of drug-assisted PHAAM in the pre-hospital setting. These rapid response vehicles were not part of this study.

The pre-hospital critical care teams covered by this study employ approximately 90 anaesthesiologists as part time pre-hospital physicians. There are no full-time pre-hospital critical care physicians in the region – all physicians primarily work in one of the five regional emergency hospitals or at the university hospital. All pre-hospital critical care physicians have in-hospital emergency anaesthesia and advanced airway management both in- and outside the operating theatre as part of their daily work. Intensive care is part of the Danish anaesthesiological curriculum.

All pre-hospital critical care teams carry the same equipment for airway management. This includes equipment for bag-mask-ventilation (BMV), endotracheal tubes and standard laryngoscopes with Macintosh blades (and Miller blades for infants and neonates), intubation stylets, *AirTraq*™ laryngoscopes, Gum-Elastic Bougies, standard laryngeal masks (LMAs), intubating laryngeal masks (ILMAs) and equipment for establishing a surgical airway. All units are equipped with a capnograph and an automated ventilator. The pre-hospital critical care teams carry a standardised set-up of medications including thiopental, propofol, midazolam and s-ketamine for anaesthesia and sedation, alfentanil, fentanyl and morphine for analgesia and suxametonium and rocuronium as neuro-muscular blocking agents (NMBAs). Lidocain is available for topical anaesthesia.

Our system has no airway management protocols or standard operating procedures (SOPs) regarding PHAAM or pre-hospital RSI [22] and the physicians use the available drugs and equipment at their own discretion.

The pre-hospital critical care anaesthesiologists in our region have an average of 17.6 years of experience in anaesthesia and on average 7.2 years of experience with pre-hospital critical care. The average pre-hospital critical care physician performs 14.5 endotracheal intubations per month, 1 of them in the pre-hospital setting.

We have previously reported details of the pre-hospital critical care physicians’ education, training, level of experience and equipment-awareness in our region [22].

We collected data from February 1^st^ 2011 until November 1^st^ 2012.

Follow-up data regarding 30-days mortality were collected in January and February 2013.

### Participants

Inclusion criteria: Consecutive patients of all ages treated with PHAAM by the participating pre-hospital critical care teams. Sollid et al. [20] define advanced airway management as any airway management beyond opening of the airway and the use of an oro-pharyngeal (*“Guedel*”) airway.

Exclusion criteria: Inter-hospital transfers.

### Variables

We collected all core data proposed and defined in the consensus-based template by Sollid et al. [20]. We would like to draw attention to the following definitions:

### Descriptive variables

We registered demographic data, patient types and indications for performing PHAAM. The indications for performing PHAAM as categorised by Sollid et al. [20] are 1) decreased level of consciousness 2) hypoxemia 3) ineffective ventilation 4) existing airway obstruction 5) impending airway obstruction 6) combative or uncooperative patient 7) relief of pain or distress 8) cardio-pulmonary arrest 9) other.

### Exposure variables

We recorded the type of PHAAM performed by the pre-hospital anaesthesiologists.

The pre-hospital critical care teams were able to perform PHAAM without the assistance of drugs, as drug-assisted PHAAM or as RSI. We defined drug-assisted PHAAM as PHAAM performed with the use of any combination of analgesic or sedative drugs without the use of a NMBA and RSI as PHETI aided by the use of any combination of a) a sedative OR an analgesic drug AND b) an NMBA.

In our study, the pre-hospital anaesthesiologists could perform PHETI by using a standard laryngoscope, the *AirTraq*™ laryngoscope or through the ILMA. They could perform PHETI with or without using a standard intubation stylet and the gum-elastic bougie. Other PHAAM techniques available were using a nasopharyngeal airway, using an SAD (LMA or ILMA) or establishing a surgical airway. We registered both the primary device used to secure a patent airway, and the use of any back-up device.

The Cormac-Lehane (CL) Score were obtained as defined by Cormac and Lehane [23].

### Endpoints and outcome variables

Primary endpoints were 1) failed PHETI 2) difficult PHETI and 3) complications related to PHAAM**.**

We defined *failed PHETI* as cases where it was not possible to establish a patent, secure airway in the pre-hospital setting.

We defined *difficult PHETI* in accordance with both the template by Sollid et al. [20] and the latest version of the *“Practice guidelines for management of the difficult airway”* by the American Society of Anesthesiologists [24] as more than one attempt needed to successfully perform tracheal intubation.

Sollid et al. [20] defines *PHAAM-related complications* as vomiting, aspiration of gastric content or blood to the lungs, accidental intubation of the oesophagus or right main stem bronchus, hypoxia (oxygen saturation < 90%), hypotension (systolic blood pressure < 90 mmHg), bradycardia (pulse <60 beats per minutes) or dental trauma. We concurred to these definitions.

The pre-hospital critical care teams or the EMTs on the road ambulances measured oxygen saturation, heart rate and blood pressure by using a LifePak 12 monitor (*Physio-Control, Redmond, USA*). End-tidal CO_2_ was monitored either via the LifePak 12 or via a Nellcor NPB-75 capnograph (*Tyco Healthcare Group LP, Pleasanton, USA*).

Secondary endpoints were the overall incidence of PHAAM-related complications linked to the number of PHETI attempts needed to secure a patent airway, the incidence of use of airway back-up devices, pre-hospital mortality and 30 days mortality.

### Data sources and data collection

We collected data from eight of the ten pre-hospital critical care teams, including the HEMS. Due to differences in organisation, staffing, case mix and caseload, the last two teams were not part of the study. The anaesthesiologists in the participating teams filled in a registration form containing all the core data recommended by Solid et al. [20]. A translated version of the registration form is available as Additional file 1. We tested the registration form for readability and user friendliness on ten randomly chosen pre-hospital critical care physicians before the study started. We did not test the registration form for inter-rater variability. Prior to the initiation of data registration, we introduced all pre-hospital critical care physicians to the registration form by e-mails and staff meetings.

Through the electronic patient journals at the hospitals in the region, we sought information regarding whether the patient survived 30-days after being treated by the pre-hospital critical care team.

We managed the data in a *Microsoft Works Database* (*Microsoft Corp.)* and crosschecked data from the registration form with data in the journal entries made by the attending physician. The primary investigator (LR) performed all data handling.

### Bias

To reduce the risk of recall bias and selection bias, the primary investigator reviewed the registration forms on a day-to-day basis. We crosschecked the registration forms with the standard pre-hospital records from the pre-hospital critical care teams to ensure the highest possible data coverage. In cases of missing data or inconsistencies, we asked the attending pre-hospital critical care anaesthesiologists to provide additional details for clarification.

### Study size

This being a descriptive study, power calculation was waived.

### Statistical methods

We analysed the data in the statistical program *Stata12 (StataCorpLP)*. Because of the rigorous crosschecking and day-to-day control, missing data were rare. If the missing data could not be obtained, we performed complete case analyses.

### Ethics

No patients had their treatment altered because of the study. All physicians participated in the study on a voluntary basis – there were no refusals. The study did not involve any alterations from normal practice and according to Danish law, it did not need the approval of the Regional Ethics Committee, nor did we need the patients’ consent to register and publish the data. The Danish Data Protection Agency approved the study (Journal number 2013-41-1462).

## Results

### Participants

**Figure 1:**
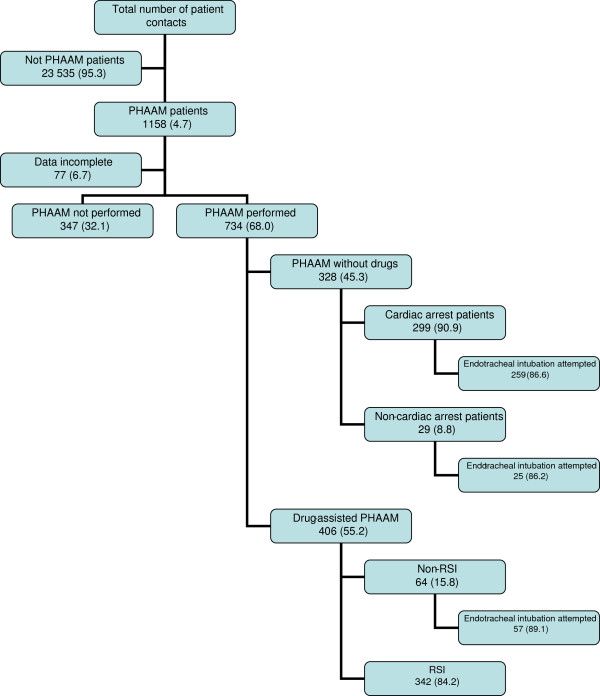
**Patient flow.** Numbers (%). PHAAM: Pre-hospital Advanced Airway Management. RSI: Rapid Sequence Intubation.

During the 21 months, the participating pre-hospital critical care teams treated 24 693 patients. The teams registered data from 734 PHAAM cases, including cases of SAD and nasopharyngeal airway usage as well as PHETI. We obtained data from 93.3% of these patients. Figure 1 is a flow diagram showing the number of patients in whom the physicians performed PHAAM, the incidence of PHAAM and PHETI performed without drugs, as drug-assisted PHAAM or as RSI.

### Descriptive data

Table 1 shows the demographics of the included patients.

**Table 1:** Demographic data

	**Number**	**%**
Total patients included (N)	734	
Age years	61.31 (median)	0 – 91 (range)
Age <16 years	42	5.7
Age < 2 years	24	3.4
Males	424	57.8
ASA-PS*-score	2.20 (median)	1–4 (range)
Pre-existing cardiac disease	142	19.3
Pre-existing hypertension	61	8.3
Pre-existing COLD**	79	10.7
Pre-existing diabetes	41	5.6
Pre-existing neurological disease	65	8.8
Other pre-existing disease	191	26.0

*American Society of Anesthesiologists Physical Status. **Chronic Obstructive Pulmonary Disease.

The average pre-hospital critical care experience among the participating doctors was 7.7 years, ranging from 1 month to 29 years.

Table 2 and Table 3 display the numbers of different types of patients and the indications for performing PHAAM. Table 4 lists the distribution of the best CL-score obtained.

**Table 2:** Patients characteristics and indication for PHAAM* (N = 734)

**Patient category**	**Number**	**%**
Isolated traumatic brain injury	40	5.4
Multitrauma (blunt)	60	8.1
Strangulation /suffocation	16	2.2
Burns	11	1.5
Other blunt trauma	16	2.2
Blunt trauma, total	143	19.5
Penetrating trauma	4	0.5
Trauma, total	147	20.0
Cardiac arrest	407	55.4
Cardiac (excluding cardiac arrest)	20	2.7
Asthma / COPD	37	5.0
Stroke / subarachnoid hemorrhage	83	11.3
Oto- rhino- laryngology	3	0.4
Other patient categories	102	13.9
First respiratory rate below 8	424	57.8
First respiratory rate over 30	24	3.3
First oxygen saturation below 90%	665 **	90.6
First heart rate below 40	352	48.0
First heart rate over 180	2	0.3
First systolic blood pressure below 90 mmHg	386	52.6
First GCS*** 3-8	640	87.2
First GCS*** 9-13	50	6.8
First GCS*** 14-15	35	4.8

*Pre-hospital Advanced Airway Management.

**279 with supplementary oxygen.

***Glasgow Coma Scale, data is missing on 9 patients.

**Table 3:** Indications for PHAAM* (n = 734)

**Indication**	Number**	**%**
Existing airway obstruction	28	3.8
Impending airway obstruction	98	13.4
Hypoxia	153	20.8
Ineffective ventilation	160	21.8
Cardiac arrest	398	54.2
Anesthesia for pain relief or distress	14	1.9
Anesthesia to combative or agitated patient	28	3.8
Decreased level of consciousness	347	47.3
Other indications	45	6.1

*Pre-hospital advanced airway management.

**Patients may have more than one indication for PHAAM.

**Table 4:** Distribution of best obtained Cormac-Lehane-score (n = 683)

**Best Cormac-Lehane-score obtained**	**Number**	**%**
1	418	61.5
2	151	22.2
3	78	11.5
4	33	4.9
>2	111	16.3

### Incidence of failed pre-hospital intubation

The pre-hospital critical care anaesthesiologists attempted PHETI in 2.8% (n = 683) of all patient contacts. Of these, 77.6% (n = 530) were successful in the first attempt (“first pass success”), 89.8% (n = 613) were successful in the first or second attempt. The overall incidence of successful PHETI was 99.7% (n = 681).

When RSI was performed (n = 345), 85.8% (n = 296) of the intubations were successful in the first attempt, 96.5% (n = 333) were successful in the first or second attempt. The overall incidence of successful PHETI following RSI was 99.7% (n = 344).

In 0.29% (n = 2) PHETI failed. One of these cases was a RSI on an adult patient with subarachnoid hemorrhage and a GCS of 3, the other case was PHETI on a 600 g neonate in cardiac arrest. The attending anaesthesiologists managed both patients with bag-mask ventilation without further PHAAM-related complications.

### Incidence of difficult intubation

More than one PHETI attempt was needed in 22.4% of the patients (n = 153). The corresponding incidence following RSI was 14.2% (n = 49).

### Incidence of PHAAM-related complications

In 14.1% of the cases (n = 104) there were complications related to PHAAM. There were no PHAAM-related deaths.

Table 5 displays the incidences of different complications related to PHAMM performed without the use of drugs, as non-RSI drug assisted PHAAM and as RSI.

**Table 5:** Complications related to PHAAM (n = 734)

**Complication**	**PHAAM without drugs**	**Drug-assisted PHAAM**	RSI*
	**(n = 328)**	**(n = 61)**	**(n = 345)**
	**Number (%)**	**Number (%)**	**Number (%)**
Vomiting / aspiration	7 (2.1)	2 (3.3)	16 (4.6)
Hypoxia	4 (1.2)	0	18 (5.2)
Bradycardia	0	0	1 (0.3)
Hypotension	1 (0.3)	0	25 (7.2)
Tube in oesophagus	14 (4.3)	2 (3.3)	15 (4.3)
Teeth trauma	0	0	0
Total**	**26 (7.9)**	**4 (6.6)**	**76 (22.0)**

*Rapid Sequence Intubation, defined as the use of at least an anaesthetic agent or an opioid AND a neuro-muscular blocking agent.

** Two patients suffered more than one complication.

Seven (38.9%) of the 18 patients who had post-RSI hypoxia had an initial oxygen saturation of less than 90%. Six (24.0%) of the 25 patients who had post-RSI hypotension had an initial systolic blood pressure of less than 90 mmHg.

The overall incidence of complications following first pass PHETI success was 7.4%. When two PHETI attempts were needed, the overall incidence of PHAAM related complications increased to 23.2%, and when more than two PHETI attempts were needed, the overall incidence of PHAAM related complications were 32.2%. Following RSI, the corresponding incidences were 11.4%, 40.0% and 20.0%.

### Other results

In 5.1% of the cases (n = 35) the anaesthesiologists used an airway management back-up device to secure a patent airway after first performing conventional laryngoscopy. Table 6 shows the methods used to solve these situations.

**Table 6:** Methods used to manage unexpected difficult intubations (n = 153)*

**Method**	**Number**	**% of all difficult intubations**
Direct laryngoscopy	116	75.8
Bag-valve-mask ventilation	2	1.3
Standard Laryngeal mask	2	1.3
Intubating Laryngeal Mask	6	3.9
Gum-Elastic-Bougie	7	4.6
Airtraq	15	9.8
Surgical Airway	1	0.7

*Not including four cases were the physician used a back-up device as first choice.

Overall pre-hospital mortality among the included patients was19.5% (n = 143). Overall mortality at 30 days was 48.9% (n = 237 out of 485. In-hospital mortality data were not available on 249 patients).

## Discussion

### Incidence of failed pre-hospital endotracheal intubation

The incidence of 0.3% failed PHETIs in our system compares to those reported from physician-staffed EMS / HEMS in the UK [6,14], Germany [12,17] and France [5].

### Incidence of difficult pre-hospital endotracheal intubation

The 22.4% overall incidence of difficult PHETI is surprisingly high. It is also higher than the overall 8.9% incidence reported from the “expert-group” of practitioners in a comparable system in Berlin [12]. It is important to notice that Breckwoldt et al. in Berlin used another definition of “difficult” PHETI than the one suggested by Sollid et al. [20]: They only counted patients with a CL-score above 2 or three or more intubation attempts as difficult. This may have led them to underestimate the incidence of difficult intubations in their system. The corresponding incidence in our system is 17.9%, which is still higher than in Berlin. We do not know the reason for this difference. The overall incidence of difficult PHETI in our study compares to the 27.1% recently reported from a US emergency department [25].

The incidence of difficult PHETI following RSI is considerably lower than in non-RSI cases and it compares to the 12.6% reported from London HEMS [14]. This is consistent with the results reported by Sakles et al. [25]. A possible explanation may be that pre-hospital RSI typically allows more time for preparations including optimal positioning of the patient before attempting PHETI compared with for instance PHETI attempts on cardiac arrest patients. Furthermore, the use of NMBAs is known to facilitate intubation success [26] and the incidence of non-RSI drug assisted PHETI in our study may be unjustifiably high. Our results suggest a need for approved first-pass success during PHETI in our system.

### Incidence of PHAAM-related complications

The overall incidence of complications in our system compares to the 14.2% reported from first–pass successful endotracheal intubations made by emergency physicians in a US emergency department [25]. We have identified no reports of overall complication rates from other physician-staffed pre-hospital services.

The current post-RSI hypoxia incidence of 5.3% in our system compares favourably to those found in other physician-staffed pre-hospital critical care systems. Helm et al from the Ulm HEMS in Germany reported an incidence of hypoxia after pre-hospital RSI of 13.3% [13], which is similar to the 10.2% reported from the Great Western Ambulance Service NHS Trust (GWAS) Critical Care Team [9], the 10.9% found in the Oslo HEMS [7] and the 18.3% reported from the London HEMS [8]. These incidences are all much lower than the 57% reported from a paramedic-based EMS in the US [27]. Possible differences in case mix and patient severity as well as in the incidence of pre-RSI hypoxia may have influenced these results, but a positive effect of the level of PHAAM training, experience and expertise among the anaesthesiologists in our system [22] probably contributed. This may also explain the relatively low 7.3% incidence of post-RSI hypotension in our system. It compares to favourably those found in the GWAS HEMS (9.7%) [9] and London HEMS (13%) [8]. The incidences of post-RSI hypoxia and -hypotension in our system are at the same level as those reported after emergency department RSI in the UK [28,29] and considerably lower than the 22% incidence of post-RSI hypotension recently reported from a US emergency department [30]. Sakles et al. reports a 9.2% incidence of hypoxia following first-pass successful endotracheal intubation in another US emergency department [25].

In our material, bradycardia following RSI rarely occurred, as was the case in GWAS HEMS [9].

Accidental intubation of the oesophagus is potentially fatal if not immediately recognised; this has been identified as a major concern in several paramedic-based EMS [31-33]. The 4.4% incidence of oesophageal intubation in our system compares to the 6.7% reported by Timmermann et al. [19]. Every single one of these misplacements were immediately recognised and corrected, presumably because of the high degree of physician experience and the routine use of capnography for tube placement confirmation. The fact that this incidence is as high as 4.4% in our system may reflect the relatively low overall incidence of first pas success.

We have identified no other papers reporting the incidence of vomiting or aspiration related to PHAAM. We may have overestimated the incidence of vomiting and aspiration as it can be unclear whether this happens before PHAAM (where it may constitute a reason for performing PHAAM) or during PHAAM.

It is important to notice that our results include patients of all age groups. Performing PHAAM on neonates and infants represents an additional challenge for the pre-hospital critical care provider. Had we restricted our analysis to adults, we may have seen a higher intubation success rate and lower complication incidences.

Our 7.4% incidence of complications in cases with first pass endotracheal intubation success compares favourably to the 14.2% reported by Sakles et al [25]. The authors show that the incidence of adverse events following endotracheal intubation rises from 14.2% following first pass success to 47.2% when two attempts were needed to secure a patent airway. Our results seem to confirm these findings. Improving the first pass PHETI success rate in our system may reduce the incidence of PHETI-related complications and improve patient safety. Possible steps towards improving first pass success may include the implementation of pre-PHETI checklists, SOPs for PHETI and a system of regular simulation-based PHAAM training as well as the use of video laryngoscopy during PHETI.

### Other results

The use of airway back-up devices in our study was more frequent than reported by Chesters et al. from the East Anglia Air Ambulance [6]. It is important to notice however, that the East Anglia Air Ambulance team use the gum-elastic bougie as a primary intubation adjunct whilst we in our system use it as a back-up device. It is noteworthy, that 75.8% of the patients with difficult PHETI in our system eventually had their tracheas successfully intubated with conventional laryngoscopy. The low surgical airway incidence in our system compares to that reported from other physician-staffed pre-hospital services [5,6,10,14].

### Limitations

Our results represent data collected prospectively in accordance with the Utstein-style template for reporting data from pre-hospital advanced airway management [20]. We report data from almost 700 patients and our data originates from eight physician-staffed pre-hospital critical care teams in a mixed rural / urban European area; this adds to the external validity of our results. The main limitation of the current study is that the attending physicians registered all the data. They are therefore subject to registration bias (systematic errors in data registration) or recall bias. The high capture rate reduces the risk of selection bias. Based on the day-to-day crosscheck of the registration forms against both the written pre-hospital journals and the compulsory entries made by the physicians in the patients’ hospital records, the extent of selection bias is probably limited. Our registrations do not allow us to differentiate between patients, who suffered post-RSI hypoxia or – hypotension because of the RSI, and patients who despite initial pre-hospital treatment were hypoxic or hypotensive when the pre-hospital critical care team initiated RSI. This may have led us to overestimate the incidence of hypotension and hypoxia caused by the RSI.

We did not design this study to detect possible longer-term complications related to PHAAM (i.e. pneumonia, acute respiratory distress syndrome or minor tracheal injuries). This may have led us to underestimate the true overall incidence of PHAAM-related complications. On the other hand, such follow-up studies may overestimate the incidence of PHAAM-related aspirations, as it would be impossible to distinguish between complications that were a result of PHAAM and those who were a result of events that happened prior to the PHAAM.

We believe that the estimated pre-hospital mortality is accurate; there were no missing data. The estimate of 30-days mortality however, carries a large degree of uncertainty as in-hospital mortality data were missing (due to a change in computer systems in the region) in 249 cases. We have no reason to believe that the mortality in the group where in-hospital mortality data are missing is different from the one in the rest of the study population, but caution is advised when interpreting these results.

### Generalisability

This was a prospective descriptional study from one homogenous Danish system of anaesthesiologist-staffed pre-hospital critical care teams. This limits the ability to generalise the findings to other systems with different staffing, caseload or case mix. Never the less, we believe that our results can be of use to other physician-staffed pre-hospital services as well as when comparing the performance and patient safety of different pre-hospital systems with different staffing.

### Perspectives

There is a need for further research on PHAAM. This also applies to the cases where PHAAM may be appropriate but is not performed. Data from these patients may provide valuable insights into the critical decision making process related to PHAAM. Furthermore, the potential effects on system performance and patient safety of introducing checklists, SOPs and guidelines for PHAAM in a physician-staffed pre-hospital system need to be established.

## Conclusion

Our anaesthesiologist-staffed pre-hospital critical care teams performed 99.7% successful pre-hospital endotracheal intubations with no fatalities related to airway management. It is important to notice however, that 22.4% of pre-hospital endotracheal intubations required more than one intubation attempt. Following RSI, the incidence of first pass success was 85.8%. The overall incidence of complications related to advanced airway management was 14.1%; this compares to that reported from emergency intubations in UK and US emergency departments. The overall post-RSI incidence of complications was 22.0%. The incidence of post-RSI hypotension was 7.3% and that of post-RSI hypoxia 5.3%. Multiple intubation attempts were associated with an increased incidence of complications and improving first pass success during pre-hospital endotracheal intubations may improve patient safety in our system.

## Abbreviations

PHAAM: Pre-hospital advanced airway management; EMS: Emergency medical service; HEMS: Helicopter emergency medical service; PHETI: Pre-hospital endotracheal intubation; CL: Cormack – Lehane; EMT: Emergency medical technician; SAD: Supraglottic airway devices; RSI: Rapid sequence intubation; LMA: Laryngeal masks; ILMA: Intubating laryngeal mask.

## Competing interests

The authors declare that they have no competing interests.

## Authors’ contributions

LR and TMH conceived the study. LR, TMH and ET designed the study. LR performed the data collection and –management. All authors contributed to data analysis and -interpretation. LR drafted the manuscript. TMH, HK and ET revised the manuscript critically for important intellectual content. All authors read and approved the final version of the manuscript for publication.

## Authors’ information

LR is a research fellow at the Norwegian Air Ambulance Foundation, consultant anaesthesiologist at the Viborg Regional Hospital and pre-hospital critical care physician in the Central Denmark Region. He currently holds the post as Programme Director for the *Scandinavian Society of Anaesthesiology and Intensive Care Medicine Advanced Educational Programme in Emergency Critical Care*. TMH is a consultant anaesthesiologist at the Aarhus University Hospital, Clinical Lead of the Pre-hospital Critical Care Team in Aarhus and pre-hospital critical care physician in the Central Denmark Region. HK is a consultant anaesthesiologist and professor of emergency medicine at the Aarhus University Hospital. ET is a consultant anaesthesiologist and professor of anaesthesiology at the Aarhus University Hospital.

## Supplementary Material

Additional file 1 This is a pdf-file containing a translated version of the registration form used by the pre-hospital critical care anaesthesiologists. Click here for file
