# Supplementary material for: Pre-hospital advanced airway management by experienced anaesthesiologists: a prospective descriptive study
Source: Scand J Trauma Resusc Emerg Med. 2013 Jul 25;21:58. doi: 10.1186/1757-7241-21-58 (PMC3733626; doi:10.1186/1757-7241-21-58)
Supplement: Additional file 1 — This is a pdf-file containing a translated version of the registration form used by the pre-hospital critical care anaesthesiologists. [file 1757-7241-21-58-S1.pdf]

Pre-hospital Advanced Airway Management in the Central Denmark Region

1) Background

Pre-hospital critical care team	☐ Aarhus<br>☐ Horsens<br>☐ Silkeborg<br>☐ Viborg	☐ Randers<br>☐ Grenå<br>☐ Lemvig<br>☐ Karup
Attending doctor a consultant	☐ Yes	☐ No
Doctor’s experience in pre-hospital critical care (years)		

2) Alarm data

Date	______________(dd.mm.yy)
Time of the alarm	_______:______
Time on scene, EMS	_______:______
Time on scene, critical care team	_______:______
Time at hospital	_______:______

3) Patient data:

Social security number	_______________________
ASA- score (prior to current event)	_______________________
Co-morbidity	

4) Patient category (more than one alternative may be checked)

Blunt trauma	☐ Traumatic brain injury<br>☐ Multitrauma<br>☐ Strangulation<br>☐ Burns<br>☐ Other blunt trauma
Penetrating trauma	☐
Non-trauma patient	☐ Cardiac arrest (CA)<br>☐ Cardiological disease (non-CA)<br>☐ Asthma / COPD<br>☐ ENT<br>☐ SAH / stroke<br>☐ Other: _______________

Leif Rognås leifrogn@rm.dk

5) First registered vital signs

Respiratory rate	________________/min
SpO_2	________________%
	With supplementary O_2 ☐
	Without supplementary O_2 ☐
Heart rate	________________/min
Systolic blood pressure	________________mmHg
Glasgow Coma Scale Score	________________

6) Indication for advanced airway management (more than one alternative may be checked)

☐ Reduced level of consciousness
☐ Hypoxia
☐ Ineffective ventilation
☐ Existing airway obstruction
☐ Impending airway obstruction
☐ Anaesthesia because of combative or uncooperative patient
☐ Anaesthesia to reduce pain or stress
☐ Cardiac arrest
☐ Other (please specify) ____

7) Airway evaluation (more than one alternative may be checked)

☐ Prior difficult airway management
☐ Severe obesity
☐ Malampati score > 2
☐ Small mouth (< 4 cm in adults)
☐ Under-bite impossible
☐ Reduced neck mobility (< 90 grader) including in-line stabilisation
☐ Short thyo-mental distance (< 3 fingers)
☐ Significant airway trauma
☐ Other (please specify) ______________________________________

☐ Airway evaluation not performed

8) ”Plan A” for airway management (only one check, please)

Spontaneous respiration

☐ No assistance (with / without supplementary O_2)
☐ With chin-lift or jaw-trust
☐ With oropharyngeal- / nasopharyngeal airway

Bag-mask-ventilation

☐ Without adjuncts
☐ With oropharyngeal- / nasopharyngeal airway

Laryngeal mask

☐ Standard
☐ Intubation laryngeal mask (”Fasttrach”)

Oral intubation

☐ Standard laryngoscopy without stylet
☐ Standard laryngoscopy with stylet
☐ Standard laryngoscopy with ”Gum - Elastic - Bougie”
☐ Airtraq

Other

☐ Nasal intubation
☐ Surgical airway

9) Best Cormack – Lehane – score (Grade 1-4) ________________

10) Numbers of airway management attempts ________________

11) Number of airway management devices used ________________

12) Final method used for airway management (only one check, please)

Spontaneous respiration

- □ No assistance (with / without supplementary O_2)
- □ With chin-lift or jaw-trust
- □ With oropharyngeal- / nasopharyngeal airway

Bag-mask-ventilation

- □ Without adjuncts
- □ With oropharyngeal- / nasopharyngeal airway

Laryngeal mask

- □ Standard
- □ Intubation laryngeal mask (”Fasttrach”)

Oral intubation

- □ Standard laryngoscopy without stylet
- □ Standard laryngoscopy with stylet
- □ Standard laryngoscopy with ”Gum - Elastic - Bougie”
- □ Airtraq

Other

- □ Nasal intubation
- □ Surgical airway

13) Post- interventional ventilations mode (only one check, please)

- □ Spontaneous ventilation
- □ Controlled ventilation
  - □ Manual ventilation
  - □ Automated ventilator
  - □ Both the above
- □ Mixed spontaneous / controlled ventilation

14) Drugs used during airway management (more than one alternative may be checked)

- □ Thiopental
- □ Propofol
- □ Midazolam
- □ S-Ketamine
- □ Fentanyl
- □ Alfentanil
- □ Morphine
- □ Suxametonium
- □ Rocuronium
- □ Ephedrine
- □ Lidocain
- □ Other (please specify)________________________________
- □ None

AKUTLÆGEBIL
REGION MIDTJYLLAND

15) Complications related to airway management (more than one alternative may be checked)

- ☐ None
- ☐ Surgical airway necessary
- ☐ Accidental intubation of the oesophagus
- ☐ Accidental intubation of right main stem bronchus
- ☐ Dental trauma
- ☐ Vomiting
- ☐ Aspiration
- ☐ Hypoxia (SpO_2 < 90%)
- ☐ Bradycardia (heart rate < 60/min.)
- ☐ Hypotension (systolic blood pressure < 90 mmHg)
- ☐ Other (please specify) ____________________

____

____

16) First post-intervention vital signs

$ETCO_2$	____________	kPa
SpO_2	____________	%
Respiratory rate	____________	/min
Heart rate	____________	/min
Systolic blood pressure	____________	mmHg

17) Vital signs upon arrival at the emergency department

$ETCO_2$	____________	kPa
SpO_2	____________	%
Respiratory rate	____________	/min
Heart rate	____________	/min
Systolic blood pressure	____________	mmHg

18) Pre-hospital outcome

- ☐ Pre-hospital death (declared dead by pre-hospital critical care team physician)
- ☐ Patient alive upon arrival at the emergency department (including patients with ongoing CPR)

19) Pre-hospital advanced airway management not performed

Did you consider performing pre-hospital advanced airway management but decided against it?

□ Yes
□ No

If yes, what was the reason? (more than one alternative may be checked)

□ Expected difficult airway management
□ Difficult access to the patient
□ Short distances to the emergency department
□ Patient's condition
□ Patient's co-morbidity
□ Lack of training / experience
□ Lack of appropriate equipment
□ Lack of necessary assistance
□ Other ______________________________

20) Comments:

Thank you very much for you collaboration!
